# Recurrent Pneumothorax with CPAP Therapy for Obstructive Sleep Apnea

**DOI:** 10.1155/2020/8898621

**Published:** 2020-12-01

**Authors:** Kartikeya Rajdev, Pretty Sara Idiculla, Shubham Sharma, Susanna G. Von Essen, Peter J. Murphy, Sabin Bista

**Affiliations:** ^1^Department of Internal Medicine, Division of Pulmonary, Critical Care & Sleep Medicine, University of Nebraska Medical Center, 42nd and Emile St., Omaha, Nebraska 68198-7400, USA; ^2^Sree Gokulam Medical College & Research Foundation, Kerala, India; ^3^University College of Medical Sciences & GTB Hospital, New Delhi, India

## Abstract

Pulmonary barotrauma such as pneumothorax (PTX) is a known complication of invasive mechanical ventilation. However, it is uncommonly reported with the use of noninvasive positive pressure ventilation (NPPV) and CPAP (continuous positive airway pressure) therapy. We present a case of a 66-year-old female who presented with chronic dyspnea on exertion secondary to right-sided diaphragmatic hernia. The patient also underwent a home sleep study which suggested obstructive sleep apnea (OSA) for which she was initiated on CPAP. She then underwent surgical repair of her right diaphragmatic hernia. The patient developed pneumothorax three times over the course of the following several months, once on the right side and twice on the left side. The patient's incidences of PTX had a temporal association with the CPAP initiation. Her CPAP therapy was discontinued permanently after the third occurrence of PTX. With this case report, we highlight the risk of barotrauma with the use of CPAP for OSA. There are very few reported cases of PTX in association with NPPV therapy for OSA. The lung-protective ventilation strategies and limiting the positive airway pressures can help reduce the risk of pulmonary barotrauma with CPAP.

## 1. Introduction

Positive pressure ventilation can be applied invasively and through noninvasive methods. Noninvasive positive pressure ventilation (NPPV) is the method of employing mechanical ventilation without endotracheal intubation, thereby eliminating the complications of invasive mechanical ventilation. It utilizes a tight-fitting nasal or facemask to deliver pressurized gas that increases the transpulmonary pressure and subsequently inflates the lungs. The process is followed by exhalation, which results from lung recoiling and expiratory muscle efforts. Continuous positive airway pressure (CPAP) is a mode where positive pressure is applied and maintained throughout the respiratory cycle to keep the upper airways patent and to increase mean airway pressure to improve oxygenation. CPAP is not strictly a ventilation method, but a broad definition of NPPV could include CPAP. CPAP is commonly utilized for the management of patients with obstructive sleep apnea (OSA). Pneumothorax (PTX) is a known complication of invasive mechanical ventilation; however, it is less common with the use of NPPV and occurrence in patients with OSA is rare. We illustrate a case of a 66-year-old female who developed recurrent PTX with CPAP therapy for OSA.

## 2. Case Report

**Figure 1:**
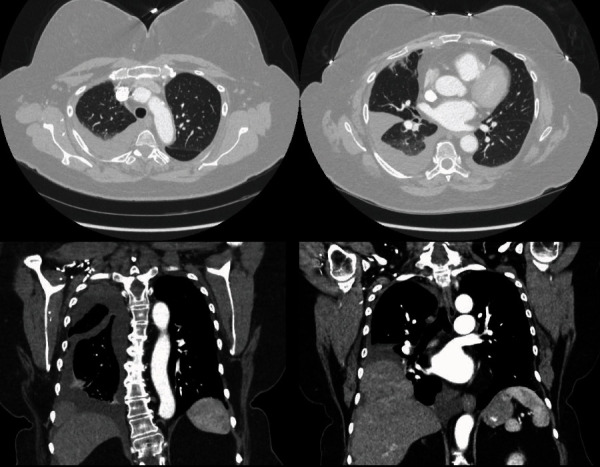
CT chest and abdomen showing right-sided pleural effusion and right lateral diaphragmatic hernia.

A 66-year-old Hispanic female, with a past medical history of hypertension and morbid obesity (BMI-40), presented to the emergency department (ED) with complaints of right flank pain of two-day duration. She also complained of dyspnea on exertion (MMRC-2) and daytime fatigue for the past 2 years. She denied any fever, cough, chest pain, nausea, or vomiting or any other symptoms. She underwent a computed tomography (CT) scan of her chest and abdomen which revealed moderate right-sided pleural effusion and a small loculated pleural effusion on the left. There were also right pleural space masses displaying associated hepatic vasculature which suggested a lateral diaphragmatic hernia containing liver (Figure 1). Thoracentesis was performed on the right side which demonstrated an exudative pleural effusion. The fluid cytology showed reactive mesothelial cells and negative for malignancy. The pleural fluid bacterial, fungal, and AFB cultures and adenosine deaminase were negative. The patient was offered surgical repair of her right-sided diaphragmatic hernia as her shortness of breath (SOB) was affecting her daily activities and exercise tolerance. Around that time, the patient also underwent a home sleep apnea test (HST) which showed mild OSA with a apnea-hypopnea index (AHI) of 13.0 per hour for a total recording time of 10 h. CPAP therapy was recommended because of potential under calling of the severity and hypoxemia that was noted out of proportion to the severity of sleep apnea. She was started on auto-CPAP with the pressure range of 4-20 cm H_2_O with ResMed Airsense 10 Auto device and nasal mask.

One month later, she underwent right diaphragmatic hernia repair through muscle-sparing thoracotomy and a wedge biopsy of the right lower lobe of the lung. Surgical biopsy showed nonspecific chronic inflammation, mild fibrosis, and intra-alveolar hemorrhage. The etiology of her pleural disease was unclear. Her postoperative period was uneventful, and a follow-up chest X-ray (CXR) demonstrated small, bilateral, residual pleural effusions with adjacent atelectasis. She was discharged home in a stable condition and resumed CPAP one week later.

**Figure 2:**
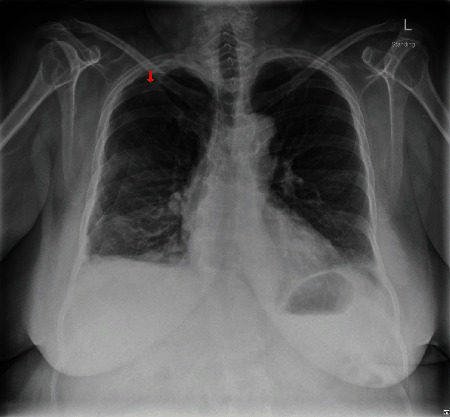
Chest X-ray showing moderate right-sided apicolateral pneumothorax (depicted by red arrow).

Five weeks after her surgery, she was seen in the clinic where she described feeling mild right-sided pleuritic chest pain and cough for one-day duration. CXR revealed a moderate right apicolateral PTX, measuring about 1.7 cm and small stable bilateral pleural effusions (Figure 2). She was managed conservatively and advised to come to the ED if her symptoms worsen. Her CPAP was temporarily discontinued. Two weeks later, she was seen in the clinic and got a CXR which showed complete resolution of the PTX. At this point, she was advised to resume her CPAP use. Her CPAP compliance report after her surgery until she developed PTX was reviewed and showed a 95^th^ percentile pressure of 14.8 cm of water and median pressure of 10.6 cm of water. The 95^th^ percentile air leak was mildly increased at 29.7 L/min (considering <24 L/min as acceptable). The residual AHI was 0.8. The patient continued to use her CPAP every night and reported improvement in her daytime fatigue and morning headaches with the use of CPAP.

**Figure 3:**
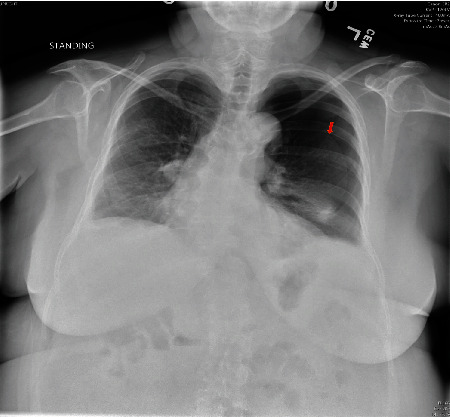
Chest X-ray showing large left-sided pneumothorax and lung collapse (depicted by red arrow).

Around 6 months after her first episode of PTX, she presented to the ED with complaints of sudden onset of SOB and left-sided chest pain. CXR showed a large left pneumothorax and lung collapse, with small bilateral pleural effusions (Figure 3). A 12 French pigtailed chest tube was placed on the left side. CT scan of the chest showed tiny residual left PTX and small bilateral layering pleural effusions with bilateral atelectatic changes. Her chest tube was removed after two days. She was discharged home and advised to discontinue the CPAP use. Two weeks later, she was seen in the clinic where she reported resolution of her symptoms, and CXR did not show any PTX. At this point, her CPAP was resumed with the same pressure settings. Her CPAP compliance report for the few months prior to her second episode of PTX showed a 95^th^ percentile pressure of 16.8 cm of water and median pressure of 11.8 cm of water. The 95^th^ percentile air leak was borderline increased at 25.9 L/min. The residual AHI was 1.1. The patient's OSA was being managed by her pulmonologist and not by a sleep specialist.

**Figure 4:**
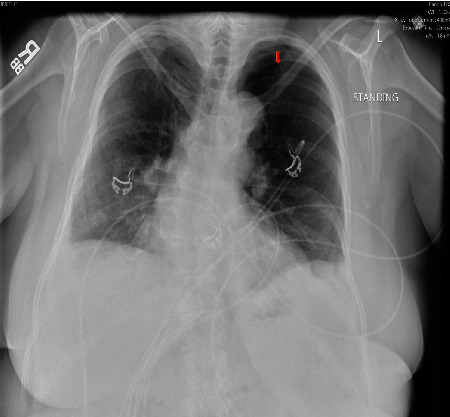
Chest X-ray showing large left-sided pneumothorax (depicted by red arrow).

Around twelve days after resuming CPAP, she presented to the ED with sudden onset of SOB. Chest imaging again revealed a moderate to large PTX on the left for which a chest tube was placed (Figure 4). The chest tube was removed after two days, and she was discharged home in a stable condition. Due to the recurrent PTX with a strong temporal association with the resumption of CPAP, it was decided to discontinue the CPAP permanently. The patient was initiated on nocturnal oxygen for nocturnal hypoxemia. The patient is currently doing well with no recurrence of PTX for the last one year.

## 3. Discussion

NPPV is a noninvasive ventilation that uses some form of an interface to ventilate the lungs. These interfaces include nasal pillow masks that fit into the nostrils, nasal masks that cover the nose, oral masks that fit into the mouth, oronasal or full-face masks that fit into both the nose and mouth, and helmet masks that cover the entire face [1]. CPAP and BPAP (bilevel positive airway pressure) are the two most commonly used types of NPPV. CPAP provides continuous pressure during the entire breathing cycle [2]. BPAP delivers preset inspiratory and expiratory pressures at alternating levels. The pressure delivered in BPAP is higher during inspiration and lower during expiration [3]. NPPV is used in the management of many clinical conditions that cause respiratory failure such as chronic obstructive pulmonary disease (COPD), asthma, thoracic wall deformity, neuromuscular diseases, obesity hypoventilation syndrome, OSA, pneumonia, pulmonary edema, and acute respiratory distress syndrome (ARDS) [4]. When applied at optimal levels to selected patients, NPPV is generally safe and well-tolerated. The side effects are usually minor, and severe complications occur very rarely. Interface-related complications include discomfort, skin rash, nasal bridge sores, nasal obstruction, claustrophobia, increased dead space, aspiration, hypersalivation, salivary retention, pressure sores, and dental problems. These are usually corrected by mask adjustment or changing the type of mask. The pressure-related complications include discomfort, ear/sinus pain, gastric insufflation, and flow-related side effects which are nasal/oral congestion, nasal/oral dryness, runny nose, nosebleed, and eye irritation. The major complications of NPPV are rare and include aspiration, mucus plugging, hypoxemia, hypotension, and barotrauma [5].

PTX is a potentially fatal complication in patients who undergo mechanical ventilation and has been reported more commonly with invasive mechanical ventilation. Pulmonary barotrauma can occur in mechanically ventilated patients as a result of a sustained increase in regional transmural pressure that causes overdistension and subsequent rupture of the alveoli. This causes air leak into the perivascular adventitia causing interstitial emphysema. Air can also dissect along the perivascular sheaths and the cervical fascial planes leading pneumomediastinum and subcutaneous emphysema, respectively. If the tension is not released, the high pressure can eventually cause rupture of the mediastinal pleura, resulting in PTX [6].

Though described mainly with invasive mechanical ventilation, the same mechanism may apply for NPPV. The application of high noninvasive positive pressures can be detrimental as it leads to high airway pressures and subsequent alveolar overdistension [7]. Due to the scarcity of literature, the exact incidence of PTX in patients who undergo NPPV is not known. It has been reported in patients who received NPPV for hypoxemia, OSA, acute respiratory failure, and chronic respiratory failure. The presence of an underlying lung condition was noted in most of the reported cases such as COPD [8–11]; cystic fibrosis [12]; Duchenne muscular dystrophy [13, 14] and chronic nonprogressive myopathy [15], pneumonia [10, 16, 17], asthma [18], and interstitial lung disease [10]; and COVID-19 [19]. The onset of PTX varied from occurring within hours of initiation to years after the initiation of NPPV therapy. After an extensive review of literature, we found only 4 other cases of PTX occurring with the use of NPPV for OSA [6, 8, 20, 21]. The characteristics of these patients with OSA and the NPPV settings, including our patient, are summarized in Table 1 and Table 2.

Imaging with chest radiograph or CT scan should be performed for patients on NPPV who present with sudden onset of dyspnea and chest pain, to evaluate for PTX. Large, symptomatic PTX requires drainage with the help of a chest tube. Choo-Kang et al. treated their case of recurrent PTX from NPPV with chemical pleurodesis using doxycycline initially followed by open pleurodesis, when the former failed [15]. The application of lung-protective strategies during mechanical ventilation can help reduce the risk of lung parenchymal injury and PTX. The protective settings for NPPV include maintaining low *P*_pl_ (<30 cm H_2_O), low tidal volume, and sound use of PEEP (positive end-expiratory pressure) to balance the distending end-inspiratory pressures as well as meet the required target of arterial oxygen pressure [7]. Since underlying lung pathologies can increase the risk of barotrauma, they should be treated appropriately. In cases that occur while on the volume-limited device, switching to a pressure-limited device is suggested especially if the PTX is slow to resolve or recur [13, 22]. This is probably because pressure-limited ventilation limits the peak airway pressure, thereby decreasing the risk of barotrauma [13].

As described above, PTX is an uncommonly reported but serious complication with the use of NPPV, and cases of PTX with NPPV use for OSA are extremely limited. In this case report, we present a case of recurrent pneumothorax secondary to CPAP therapy for OSA, with a strong temporal association with the initiation of CPAP. There has not been a consensus on the best treatment modality for mild OSA due to limited evidence base, and many physicians would offer CPAP to patients with mild OSA. The American Academy of Sleep Medicine (AASM) recommends offering positive airway pressure therapy to all patients who have been diagnosed with OSA [23, 24]. They defined OSA as either a respiratory disturbance index (RDI) ≥ 15 events per hour with or without symptoms or an obstructive RDI between 5 and 14 events per hour that is accompanied by any of the following: fatigue, nonrestorative sleep, sleepiness, or insomnia symptoms; habitual snoring and/or breathing interruptions; waking up with gasping, breath-holding, or choking; and hypertension, cognitive dysfunction, mood disorder, coronary artery disease, congestive heart failure, stroke, type 2 diabetes, or atrial fibrillation [25]. The obstructive RDI is defined as the number of obstructive apneas, obstructive hypopneas, and respiratory effort related arousals (RERAs) per hour of sleep. A recent randomized clinical trial by Wimms et al. found an improvement in the quality of life in patients with mild OSA treated with CPAP [26]. That being said, we believe that our patient's severity of OSA on the HST was underestimated as the total recording time was 10 h, whereas the usual sleep duration as reported by the patient and the average CPAP use on the compliance report was around 7-8 h. This is because the HST measures RDI over a total recording time, unlike in-laboratory study which uses total sleep time. The literature supports that the treatment provided by sleep and nonsleep specialists resulted in a similar quality of life, symptom scores, and adherence [27]. However, with this article, we would like to highlight that the involvement of a sleep specialist in patients with a complicated course of OSA treatment may be useful. A high air leak can lead to the generation of high CPAP pressures. Therefore, air leak should be addressed, especially in patients who are mouth breathers and are on a nasal mask. An in-laboratory titration study can be pursued to determine the optimal positive pressure level required to normalize the AHI (<5) in order to help limit the positive pressures. Nocturnal oxygen therapy for OSA significantly improves oxygen saturation and intermittent hypoxia in patients with OSA [28]. However, with the current evidence, it is difficult to recommend oxygen therapy over CPAP for the treatment of patients who are unable to tolerate CPAP therapy. Oxygen therapy has been shown to prolong the duration of apnea and hypopnea events, perhaps as a result of suppressing hypoxic respiratory drive which can potentially worsen acidosis [28]. It also does not address the upper airway obstruction that is central to the pathophysiology of OSA. Oral appliances like mandibular advancement devices, weight loss, positional therapy, and upper airway surgical options are some of the alternatives that can be utilized in patients who are not able to tolerate positive airway pressure therapy.

In conclusion, health-care providers should be aware of pneumothorax as a potential complication from the use of NPPV for OSA and limiting the positive airway pressures or use of alternative therapy when feasible might help decrease the risk of such complications. A timely referral to a sleep specialist may be warranted in managing CPAP-related complication while treating OSA.

## Figures and Tables

**Table 1:** Characteristics of patients who developed pneumothorax with NPPV therapy for OSA.

Case	Age (years)	Sex	NPPV indication	Underlying lung pathology	NPPV type	NPPV pressure settings
Current case—Rajdev et al.	66	F	Obstructive sleep apnea	Right-sided pleural effusion and diaphragmatic hernia	CPAP with nasal mask	(i) Auto-CPAP pressure-4-20 cm H_2_O
Langner et al. [6]	68	NA	Obstructive sleep apnea	None	CPAP	(i) Initial therapy pressure-9 cm H_2_O (ii) Pressure later increased-13 cm H_2_O
Herrejón Silvestre et al. [8]	67	M	Obstructive sleep apnea	Pan acinar emphysema	BPAP with nasal mask	(i) Inspiratory pressure-10 cm H_2_O (ii) Expiratory pressure-5 cm H_2_O
Mao et al. [20]	64	M	Obstructive sleep apnea	None	PAP (ASV)	(i) Pressure support-5-10 cm H_2_O (ii) EPAP-15 cm H_2_O
Chicote Álvarez et al. [21]	72	NA	Obstructive sleep apnea	NA	CPAP	(i) NA

ASV: adaptive support ventilation; BPAP: bilevel positive airway pressure; CPAP continuous positive airway pressure; EPAP: expiratory positive airway pressure; F: female; H_2_O: water; M: male; NA: not available; NPPV: noninvasive positive pressure ventilation; OSA: obstructive sleep apnea.

**Table 2:** Features of PTX in the affected patients.

Case	First PTX onset after NPPV	PTX frequency	Intervention for PTX
Current case—Rajdev et al.	2 months	Three times	Chest tube
Langner et al. [6]	Years	Once	Chest tube
Herrejón Silvestre et al. [8]	Within hours	Once	Chest tube
Mao et al. [20]	NA	Twice	Chest tube
Chicote Álvarez et al. [21]	NA	Once	Chest tube

NA: not available; NPPV: noninvasive positive pressure ventilation; PTX: pneumothorax.
